# The left ventricle in aortic stenosis – imaging assessment and clinical implications

**DOI:** 10.1186/s12947-015-0017-4

**Published:** 2015-04-29

**Authors:** Andreea Călin, Monica Roşca, Carmen Cristiana Beladan, Roxana Enache, Anca Doina Mateescu, Carmen Ginghină, Bogdan Alexandru Popescu

**Affiliations:** Department of Cardiology, University of Medicine and Pharmacy ”Carol Davila”, Euroecolab, Bucharest, Romania; Institute of Cardiovascular Diseases ”Prof. Dr. C. C. Iliescu”, Sos Fundeni 258 sector 2, 022328 Bucharest, Romania

**Keywords:** Aortic stenosis, Left ventricular function, Imaging

## Abstract

Aortic stenosis has an increasing prevalence in the context of aging population. In these patients non-invasive imaging allows not only the grading of valve stenosis severity, but also the assessment of left ventricular function. These two goals play a key role in clinical decision-making. Although left ventricular ejection fraction is currently the only left ventricular function parameter that guides intervention, current imaging techniques are able to detect early changes in LV structure and function even in asymptomatic patients with significant aortic stenosis and preserved ejection fraction. Moreover, new imaging parameters emerged as predictors of disease progression in patients with aortic stenosis. Although proper standardization and confirmatory data from large prospective studies are needed, these novel parameters have the potential of becoming useful tools in guiding intervention in asymptomatic patients with aortic stenosis and stratify risk in symptomatic patients undergoing aortic valve replacement.

This review focuses on the mechanisms of transition from compensatory left ventricular hypertrophy to left ventricular dysfunction and heart failure in aortic stenosis and the role of non-invasive imaging assessment of the left ventricular geometry and function in these patients.

## Introduction

Aortic stenosis (AS) is the third most common cardiovascular disease in Western countries and the main indication for valve replacement in adult patients [1]. The assessment of AS severity, symptomatic status and left ventricular (LV) systolic function have the key role in patients' management algorithm [2,3].

Left ventricular ejection fraction (LVEF) is currently the only LV function parameter that guides intervention in asymptomatic patients with severe AS [2]. Even in patients with AS and preserved LVEF current imaging techniques allow the detection of subtle LV structural and functional changes that might alter the long-term prognosis [4,5]. In addition, some of the patients with normal LVEF have reduced transvalvular flow rate that entails significant challenges with regards to evaluation and clinical decision-making [6]. This review focuses on the mechanisms of progression from compensatory LV hypertrophy (LVH) to LV dysfunction and heart failure (HF) in AS and on specific issues regarding the noninvasive imaging assessment of LV structure and function in these patients.

### Left ventricular hypertrophy as a compensatory mechanism in aortic stenosis

Concentric hypertrophy is the main compensatory mechanism for pressure overload [7] in patients with significant AS. The increase of contractile elements leads to increased contractile force and reduces systolic wall stress. Thus, despite very high intraventricular systolic pressure, cardiac output and filling pressures may remain within normal limits.

Although valvular obstruction is the most important stimulus for LVH in patients with AS, the LV response depends not only on the severity of AS, but also on a multitude of individual factors. Age, gender, genetic variation in the renin–angiotensin system, co-existing coronary artery disease, hypertension, or significant associated aortic regurgitation are additional factors influencing the response of the LV to increased valvular load [8-12].

In patients with concomitant systemic hypertension, an increase in blood pressure superimposed on an increased valvular resistance leads to a significant increase in LV systolic wall stress. The contribution of vascular load is essential to be recognized in all patients with AS, as it further increases LV global afterload, which is associated with decreased stroke volume, impaired myocardial function and reduced survival [13].

### Pathophysiology of left ventricular dysfunction in aortic stenosis

If the valvular obstacle is not removed, the adaptive mechanisms to pressure overload are exceeded, either because the limit of sarcomere extension is reached or because marked LVH with increased diastolic stiffness prevents adequate LV filling [14]. The LV becomes unable to maintain a normal stroke volume in the setting of limited preload reserve, a condition known as "afterload mismatch". Consequently, systolic wall stress becomes markedly elevated, and LVEF decreases. Left ventricular dilation might be present in this late phase, with eccentric LV remodelling or hypertrophy. In the absence of significant coronary lesions, reduced LVEF in AS occurs only in end-stage disease, and is usually preceded by symptom occurrence. Nevertheless, cardiac events may occur in patients with AS before the decline of LVEF. The afterload mismatch state definition implies that myocardial contractility is not depressed, and the relief of valvular obstruction will allow an efficient recovery of the LV in terms of size and function [14]. An improvement of LV systolic function is observed in most of these patients after successful AVR [15]. However, diastolic dysfunction and an abnormal response to exercise may persist for several years after AVR, even in patients with normal LVEF [16].

A large number of experimental and clinical studies addressed the transition from the "compensated state" of LVH to overt HF in AS and proposed a series of potential underlying mechanisms. Interstitial myocardial fibrosis, myocyte degeneration, and apoptosis are early structural changes in patients with severe AS, their extent being related to increasing LV filling pressures and decreased LVEF [17].

Impaired coronary flow reserve and inadequate subendocardial blood flow are found in patients with AS even in the absence of significant coronary artery disease [18]. These are related to the severity of AS, haemodynamic load on the LV, and reduced diastolic perfusion time, rather than to the increase in LV mass [18] and may represent the substrate for LV longitudinal dysfunction. This is an early finding in patients with significant AS even in the presence of a normal LVEF [5]. Compensatory changes in LV geometry with increased relative wall thickness (RWT) and preserved radial and circumferential function, mainly determined by mid-wall myocardial fibers, may explain the preservation of LVEF in these patients [5].

Diastolic dysfunction has an important role in the pathogenesis of symptoms and the progression to HF in patients with AS [19]. It appears early in the disease process as the result of LVH and interstitial fibrosis [17] with subsequent impaired relaxation and increased LV chamber stiffness.

### Noninvasive assessment of left ventricular function – specific issues in aortic stenosis

An accurate assessment of LV remodelling and function is warranted in all patients with significant AS. The identification of early signs of impaired myocardial contractility is particularly important in asymptomatic patients with severe AS. A series of new noninvasive imaging derived parameters of LV function emerged as predictors of disease progression in AS.

#### Echocardiography - conventional measurements and additional parameters of LV function

**Figure 1:**
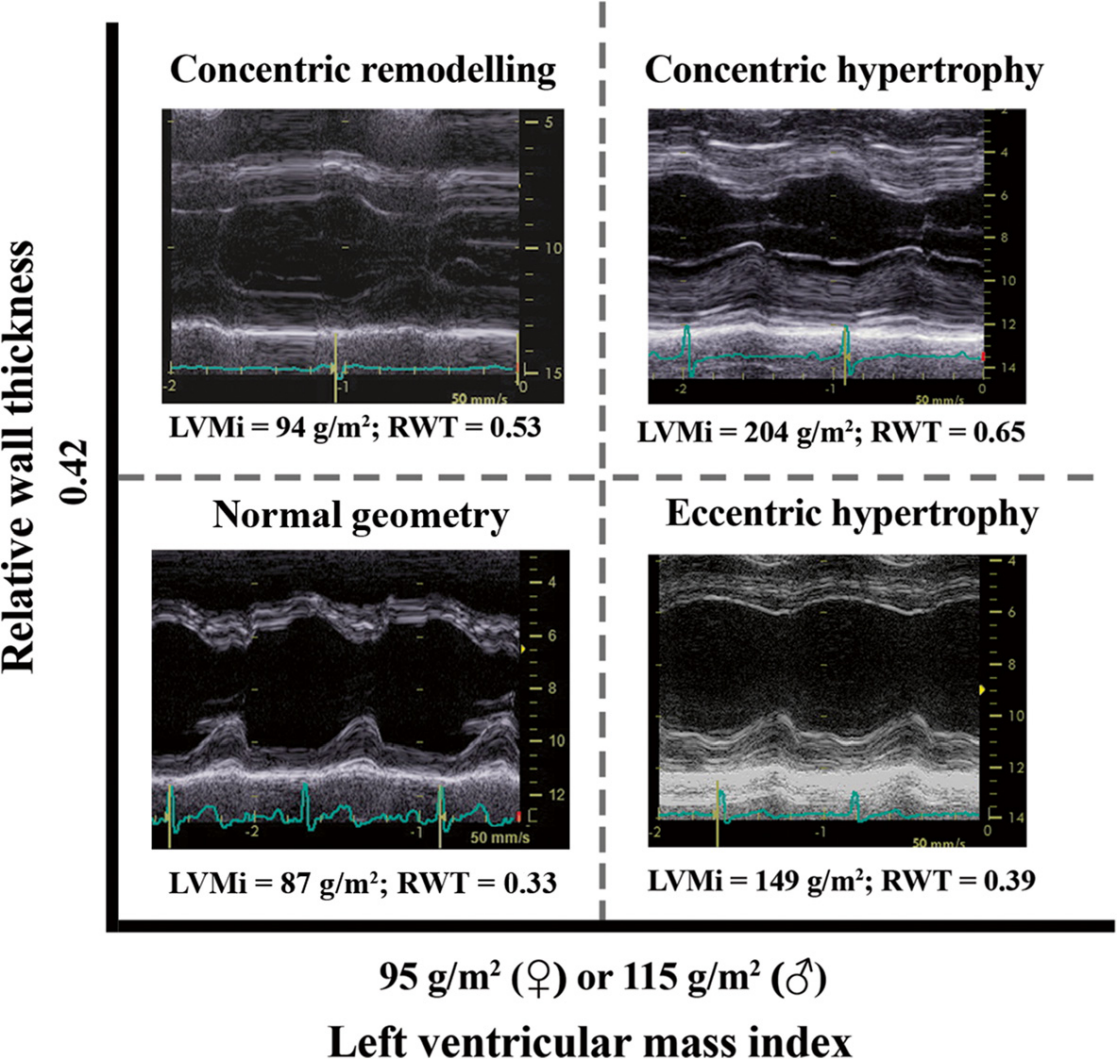
Classification of LV geometry type based on relative wall thickness (RWT) and left ventricular mass index (LVMi). Each type of abnormal LV geometry is illustrated by M-mode images obtained in patients with severe AS.

Echocardiography remains the investigation of choice both for the assessment of AS severity and LV function. Linear LV dimensions should be measured in all patients based on existing recommendations [20] for further estimation of LV mass and RWT, in order to classify the type of LV remodelling (Figure 1). The currently accepted concept of LVH is based on data obtained using conventional echocardiography for the assessment of LV mass.

Three-dimensional echocardiography overcomes the inherent geometrical assumptions from 2D echocardiography and its accuracy in measuring LV volumes and mass was already demonstrated against cardiac magnetic resonance imaging (CMR) [21], but lack of specific cut-off values hampers its use in clinical practice.

Conventional echocardiography allows the estimation of LV systolic function by measuring LV endocardial and midwall fractional shortening, LVEF, mitral annular plane systolic excursion (MAPSE), LV stroke volume and myocardial performance index. Both LV endocardial shortening fraction and LVEF are derived by measuring endocardial displacement, and both overestimate systolic function in the presence of concentric LVH. Mitral annular plane systolic excursion reflects global LV longitudinal systolic function and is a more sensitive marker of systolic dysfunction compared to LVEF. It declines with increasing AS severity independent of LVH, implying a direct relation with the increased haemodynamic load [22]. Decreased MAPSE is related to increased subendocardial fibrosis and a cut-off value of 9 mm had an excellent accuracy to distinguish between moderate and severe AS [23]. This might be particularly useful in the challenging clinical scenario of low-gradient severe AS with preserved LVEF, in order to differentiate patients with truly severe AS from those with moderate AS.

Accurate calculation of LV stroke volume (using LV outflow tract time-velocity integral and diameter) must be included in the echocardiographic evaluation of patients with AS, especially in patients with severe AS (based on aortic valve area calculation), preserved LVEF (>50%) and a low transvalvular gradient (mean gradient < 40 mmHg). A cut-off value of < 35 ml/m^2^ is an essential criterion for the definition of paradoxical low-flow AS [3,13]. These patients have typical echocardiographic aspects including a small LV cavity size, impaired LV filling, reduced arterial compliance and elevated valvulo-arterial impedance reflecting a higher LV global load [13]. Recently published data revealed that many of these patients have a severe AS when using aortic valve weight as a reference method [24].

Although LVEF below 50% is the only parameter of LV function that guides intervention in patients with severe AS [2], it is not a good measure of myocardial contractility. Moreover, LVEF is mainly determined by radial function, which can be normal for a long time, even in the presence of subendocardial fibrosis [23].

A separate analysis of the various components of LV deformation by current echocardiographic techniques allows a better understanding of progression to HF in patients with AS and an early detection of asymptomatic patients who are most likely to benefit from more aggressive intervention.

A series of clinical studies based on *tissue Doppler imaging* (TDI) assessed subtle changes in LV function in patients with significant AS and preserved LVEF. Peak systolic annular velocities (S') were significantly reduced in non-ischemic patients with moderate and severe AS, in the presence of normal LVEF and cardiac index [4,25]. Longitudinal systolic strain and strain rate parameters were also significantly decreased in these patients, their decline being related to the severity of AS [26]. A rapid improvement of these parameters was demonstrated after aortic valve replacement (AVR), before any significant changes in LV mass and LVEF, suggesting that they partially depend on the presence of LV afterload [26,27].

Two-dimensional speckle-tracking echocardiography (2D-STE) allows a multidirectional angle-independent evaluation of myocardial deformation providing a comprehensive assessment of LV function [28]. The results from clinical studies using 2D-STE in patients with severe AS and preserved LVEF confirmed the significant decrease in LV longitudinal strain [5,29-31] (especially in the basal segments) and showed the impact of reduced longitudinal deformation on exercise capacity and prognosis in asymptomatic patients [5]. More cardiac events were observed during follow-up in patients with lower values of longitudinal strain in the LV basal segments (below −13%), while a global longitudinal strain (GLS) below −18% predicted an abnormal exercise response with a sensitivity of 68% and a specificity of 77% [5]. Average longitudinal strain depends not only on AS severity but also on the type of LV remodelling, with lower values in patients with higher LV mass and RWT [29].

A gradual impairment in longitudinal, circumferential, and radial deformation was found in a large number of patients with a wide range of AS severity, suggesting a progressive subendocardial to transmural impairment of myocardial function with increasing LV afterload [30]. In asymptomatic patients with severe AS all three components of myocardial deformation were more impaired in patients with higher global LV afterload and in patients with a low stroke volume index [31]. The authors suggested that a decrease in circumferential function in patients with AS may be a marker of an advanced stage of the disease and could identify patients at higher risk, particularly when it is associated with a low-flow state.

However, data regarding circumferential and radial LV deformation are not consistent between studies [5,31], in part because of a higher variability of these parameters.

**Figure 2:**
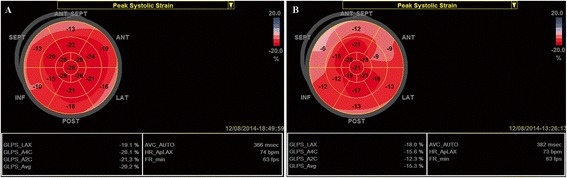
Left ventricular global longitudinal strain (GLS) measured by speckle tracking echocardiography in two asymptomatic patients with severe AS, a similar degree of concentric LVH and LVEF > 60%. Reduced values of longitudinal deformation in the basal LV segments are observed in the first patient, but with a GLS value within normal range (−20%) **(A)**. Impaired GLS (−15%) was found in the second patient, with more severely reduced values of longitudinal deformation in the basal segments **(B)**. Stress echocardiography was performed in both patients. The second patient experienced dyspnea at a low level of exercise while the first remained asymptomatic. Angiography in the second patient revealed no significant coronary lesions.

On the other hand, GLS seems to be a more robust parameter and emerged as a potentially useful tool in the assessment of subclinical LV dysfunction in AS (Figure 2).

Echocardiographic parameters of longitudinal LV function (such as MAPSE and GLS) allow an indirect assessment of fibrotic changes in patients with AS. They are surrogate markers of the presence and severity of myocardial fibrosis and are superior to LVEF in the assessment of latent LV dysfunction. Although myocardial reflectivity is directly related to myocardium collagen content and can be quantitatively assessed using ultrasonic backscatter signal [32], this technique is not widely used in clinical practice.

Left ventricular torsional deformation has an important role in both LV ejection and filling [33,34]. An increased apical rotation leading to an increased LV torsion was demonstrated by 2D-STE in symptomatic patients with severe AS and preserved LVEF [35,36], with a normalization of these parameters 6 months after AVR [36]. Delayed LV diastolic untwisting, significantly related to increased LV filling pressures, was also reported in these patients [35].

The assessment of LV diastolic function in patients with AS should be performed using the existing recommendations [37] and taking into account the limitations imposed by different associated conditions (e.g. mitral annular calcification, significant mitral or aortic regurgitation, atrial fibrillation). Reduced values of mitral annulus early diastolic myocardial velocity (e') and higher values of E/e' ratio were found in asymptomatic patients with moderate AS when compared to controls indicating an early impairment of diastolic function [38]. In patients with moderate to severe AS, E/septal e' ratio was validated for the estimation of LV filling pressures against cardiac catheterization, a value ≥13 identifying an LV end-diastolic pressure >15 mmHg with good accuracy [4].

Doppler transthoracic or transoesophageal echocardiography, usually with intravenous adenosine infusion, allow the noninvasive evaluation of coronary flow reserve, a surrogate for the coronary microcirculation in patients with normal angiographic coronary arteries. Most studies conducted in patients with significant AS showed that coronary flow under resting conditions is significantly higher and hyperemic flow velocity is lower compared with controls [39].

The current recommendations for stress echocardiography in patients with AS are still limited [2,3]. The assessment of LV contractile reserve using low-dose dobutamine stress test has clear prognostic implications in patients with low flow low gradient AS and reduced LVEF [40]. The evaluation of LV function during exercise can also provide incremental prognostic information in asymptomatic patients with severe AS. A decrease or a limited increase in LVEF at exercise is associated with a markedly reduced midterm cardiac event-free survival [41]. Moreover, the assessment of GLS at exercise is able to more accurately detect latent LV systolic dysfunction compared to changes in LVEF [42].

The evaluation of vascular afterload is a useful additional diagnostic tool in patients with AS. It was demonstrated that both carotid and aortic stiffness are associated with increased LV filling pressures, plasma BNP and symptoms in patients with moderate and severe AS [43]. Moreover, increased aortic rigidity is independently related to impaired longitudinal LV function in patients with severe AS and preserved LVEF [44]. The study of these parameters may identify patients at a more advanced stage of the disease, although their independent prognostic value awaits confirmation in larger prospective studies. Conversely, no relationship was demonstrated between pulse wave velocity, the most widely used parameter of arterial stiffness, and LVEF, in patients with significant AS undergoing AVR [45].

Valvulo-arterial impedance, which provides an estimate of the global LV haemodynamic load, is superior to the standard indexes of AS severity in predicting LV dysfunction. Its independent prognostic value was already demonstrated in both symptomatic and asymptomatic patients with significant AS [13,46].

#### Cardiac magnetic resonance imaging

In patients with AS, CMR allows the quantification of the severity of valve disease, provides additional information regarding the enlargement of the ascending aorta and the consequences of pressure overload on LV structure and function.

There has been growing interest in the assessment of myocardial fibrosis by CMR. The development of diffuse fibrosis emerged as a key mechanism for the progression to HF of patients with AS [17] and a potential treatment target [47]. By the use of equilibrium contrast CMR an increased level of diffuse myocardial fibrosis was found in patients with severe AS awaiting surgery [48], but with a considerable overlap between patients and controls. The degree of diffuse fibrosis was not correlated to LV mass or LVEF, but it was the strongest determinant of functional status at baseline.

**Figure 3:**
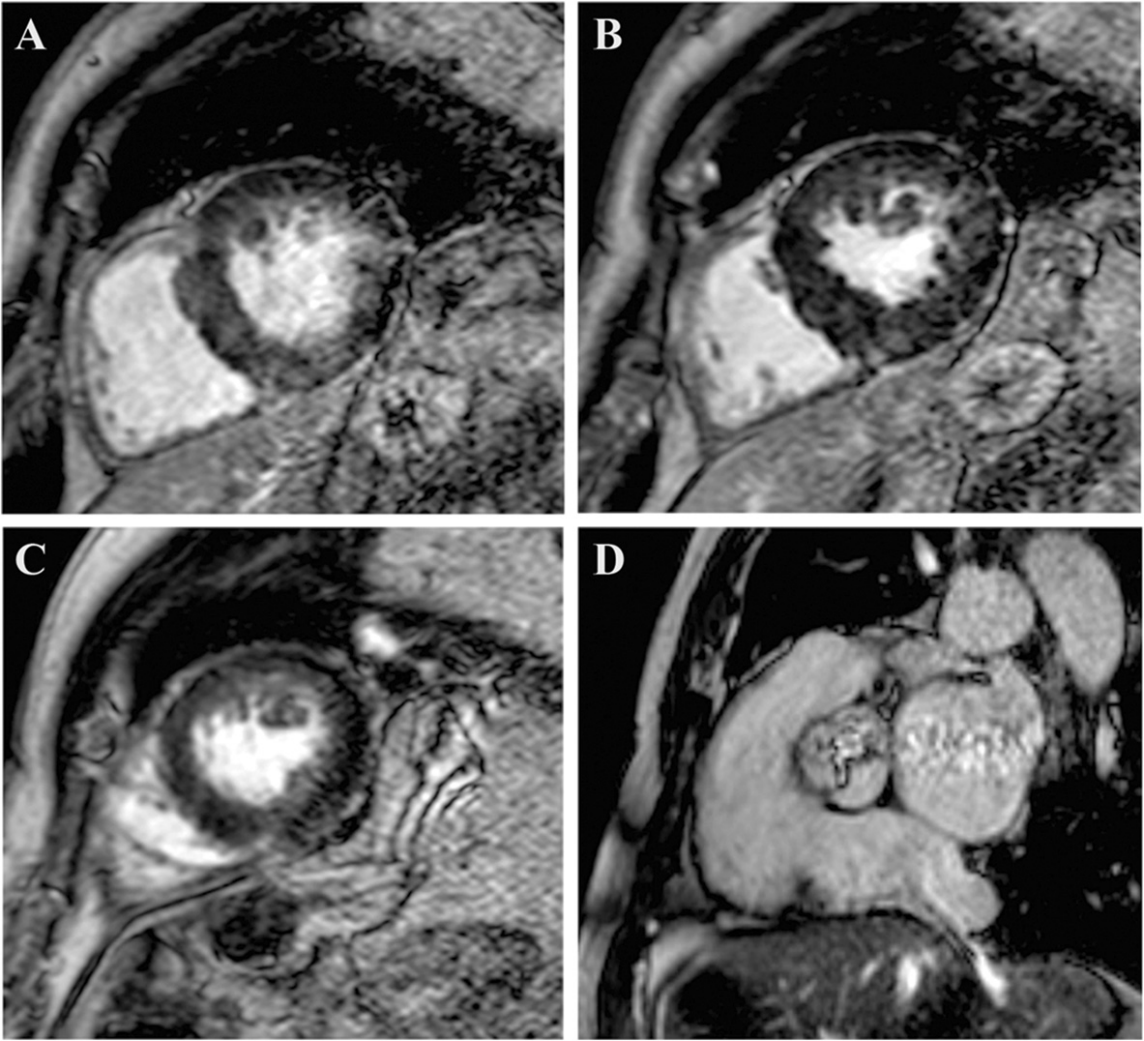
Late gadolinium enhancement (LGE) CMR in basal to apical short axis **(A-C)** and systolic frame Cine CMR through the aortic valve **(D)** in a patient with severe AS. Patchy diffuse LGE may be observed **(A-C)** together with a severely reduced valve area **(D)**. Courtesy of Dr Anca Florian, Dept. of Cardiology, Uniklinikum Muenster, Germany.

Late gadolinium enhancement (LGE) CMR is the gold standard imaging method for assessing focal, replacement fibrosis [48] (Figure 3). In symptomatic patients with severe AS myocardial replacement fibrosis is found mainly in the subendocardial layer of the LV and its degree decreases from the base to the apex [49,50]. Its presence was associated to decreased LV longitudinal function and poor postoperative outcome [49]. A peak systolic longitudinal strain of less than −11.6% has a sensitivity of 65% and a specificity of 75% for predicting myocardial fibrosis (defined as LGE >10%) [50].

Patients with paradoxical low-flow low gradient AS have a higher degree of myocardial fibrosis and a more impaired LV longitudinal function when compared to patients with normal flow high gradient AS [23]. These changes may contribute to their reduced LV stroke volume and transvalvular gradient and the worse outcomes [13,23,24].

The assessment of myocardial fibrosis by CMR may in the future refine the selection of asymptomatic patients with severe AS, who may benefit from early intervention, although this requires confirmation in larger prospective studies.

Subclinical deterioration of LV function can also be assessed by CMR, which is considered the reference standard for the assessment of myocardial deformation [51]. A few clinical studies using tagged CMR demonstrated for the first time that LV torsion is increased, and LV untwisting is delayed in patients with significant AS [52].

Given its complexity, high cost and limited availability, the utility of CMR for the assessment of LV strain in AS remains confined to research in certain academic centers.

Myocardial perfusion reserve may also be assessed by CMR and is independently associated with objectively measured exercise capacity in patients with severe AS [53].

#### Computed tomography

Multi-slice cardiac computed tomography (CT) offers additional data regarding the ascending aorta and the LV outflow tract in patients with AS, and is useful in quantifying the valvular and coronary calcification, with specific application in patients who are eligible for a transcatheter AVR [2]. This technique may also be used to exclude coronary artery disease (CAD) in younger patients with AS who are at low risk of atherosclerosis [2]. However, invasive coronary angiography is strongly recommended when CAD is a concern.

Although the assessment of LV volumes and global function by cardiac CT has a wider availability when compared to CMR, its utility in patients with AS is not established in clinical practice.

Positron emission tomography allows the noninvasive quantification of the transmural distribution of myocardial blood flow. Myocardial flow reserve can also be evaluated using dynamic perfusion imaging at rest and during dipyridamole stress. This parameter is more severely impaired in the subendocardial layers of the LV in patients with LV hypertrophy atributable to severe AS and angiographically normal coronary arteries. In patients with low-flow, low-gradient AS a higher resting myocardial blood flow and a reduced flow reserve (linked to the AS severity) were also found using this technique [54].

The comparative role of currently used noninvasive imaging techniques in the assessment of LV structure and function in AS is illustrated in Table 1.

**Table 1:** **Role of noninvasive imaging techniques in the assessment of the left ventricle in patients with aortic stenosis**

	**Advantages**	**Disadvantage/Pitfalls**
**Echocardiography**		
LV geometry parameters (LV mass and RWT)	- mandatory for classification of LV remodelling	- less accurate and reproducible estimation of LV mass compared to CMR, in particular in patients with large left ventricles
	- easy to perform	
	- demonstrated prognostic value	
LV ejection fraction	- established prognostic value in patients with AS	- overestimates LV systolic function in this setting
	- practical implications in the decision making process	- difficult to measure in patients with suboptimal acoustic window
MAPSE	- widely available and easy to measure	- problematic in patients with mitral annular calcification
	- useful for the detection of LV longitudinal dysfunction	
Peak systolic myocardial velocity (by TDI)	- early marker of LV dysfunction especially when assessed during or after exercise in patients with asymptomatic AS	- angle dependent
		- does not reflect global LV function in pts with segmental wall motion abnormalities
STE derived global longitudinal strain	- relatively easy to obtain parameter quantifying longitudinal LV systolic function	- requires good image quality and dedicated software
	- recent data support its prognostic value in AS patients	- lack of standardization on different echo machines (inter-vendor variability)
Parameters reflecting LV diastolic function	- allow noninvasive estimation of LV filling pressures	- less accurate in patients with associated mitral annular calcification and/or significant mitral regurgitation
	- impaired diastolic function is associated with symptomatic status in severe AS	
**Cardiac magnetic resonance imaging**	- gold standard assessment of LV volumes, mass and EF as well as myocardial deformation	- high cost and limited availability
	- allows the detection and quantification of interstitial and focal myocardial fibrosis - demonstrated prognostic value in AS	- adverse reactions after i.v. administration of gadolinium-based contrast agents
		- results from LGE method vary between different imaging studies (less suitable for folow up studies)
		- the equilibrium contrast method for the assessment of diffuse fibrosis is still complex and time-consuming
**Computed tomography**	- allows the assessment of LV volumes and global LV function	- exposure to radiation and potential contrast nephrotoxicity
	- wider availability when compared to CMR	- limited data regarding LV function assessment in AS patients

### Concomitant conditions that may influence the assessment of LV function

Varying degrees of mitral regurgitation (functional or organic) are often found in patients with severe AS. Concomitant severe mitral regurgitation may lead to an overestimation of LVEF and LV fractional shortening.

Left ventricular remodelling is a determinant of functional mitral regurgitation not only in patients with AS and reduced LVEF but also in patients with preserved LVEF. A significant inverse correlation was found between effective regurgitant orifice area and LV systolic longitudinal shortening in these patients, suggesting that subclinical LV dysfunction might compromise proper mitral valve function [55].

**Figure 4:**
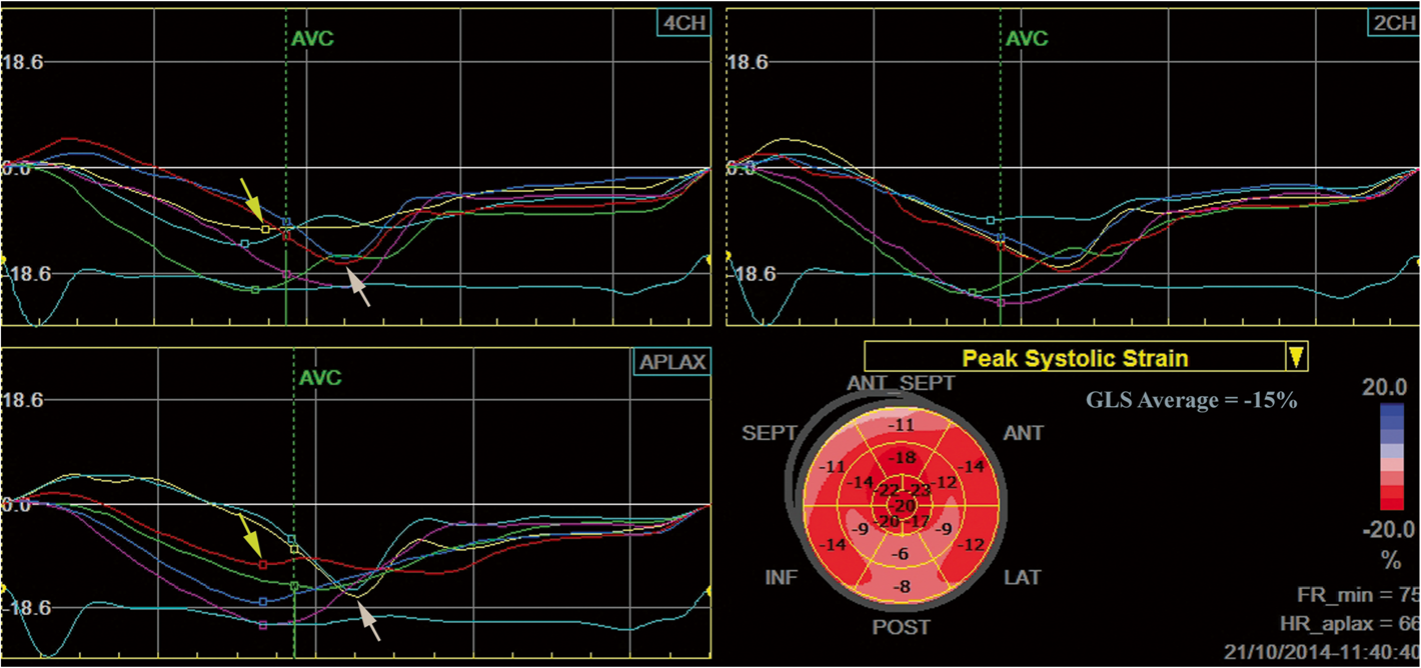
Left ventricular longitudinal strain measured by speckle tracking echocardiography in a patient with severe aortic stenosis and chest pain. A nonuniform reduction of longitudinal deformation can be observed, with reduced values of peak systolic strain in the basal segments of the interventricular septum (yellow arrows) and post-systolic shortening in mid and basal segments of the lateral wall (white arrows). Coronary angiography revealed a calcified left main stenosis (80%) extended to the origin of the circumflex artery and a hypoplastic right coronary artery.

Significant CAD is present in more than 50% of patients with severe AS who are over 70 years [56]. A lower LVEF and a more impaired longitudinal and radial LV function were found in patients with AS in the presence of concomitant CAD [57] (Figure 4). These patients have a worse prognosis, more post-operative morbidity and increased mortality related to the effects of pre-existing ischemic myocardial damage and comorbidities [58].

### Prognostic significance of left ventricular remodelling in aortic stenosis

Current guidelines strongly recommend AVR in all patients with severe AS when associated with either symptom related to AS or an abnormal LVEF (<50%) [2]. In clinical practice, most asymptomatic patients with severe AS have a preserved LVEF and require a careful risk stratification in order to choose between early elective intervention and watchful waiting. Efforts are being made to anticipate the onset of symptoms in patients with severe AS and identify early signs of myocardial dysfunction.

In symptomatic patients with severe AS, AVR is firmly indicated but further risk stratification may be needed in patients with severely impaired LV function or extensive comorbidities, especially with the recent extent of transcatheter AVR interventions.

The prognostic value of LV dysfunction in patients with AS was demonstrated by Lund *et al*. [59] in symptomatic patients with AS undergoing AVR. Left ventricular ejection fraction <60% and diastolic dysfunction assessed by radionuclide ventriculography were the only independent risk factors for early death.These results were further confirmed by more recent data, LVEF being identified as an independent predictor of all-cause mortality in patients with significant AS, irrespective of the presence of symptoms [60].

Beyond LVEF, excessive LVH and abnormal TDI parameters of LV function are cited by the current guidelines [2] as predictors of symptom development and adverse outcomes in asymptomatic patients with AS, although specific cut-off values are not provided. Inappropriately high LV mass was associated with a 4.5-fold higher risk of adverse events in asymptomatic patients with severe AS [61]. On the other hand, in symptomatic patients, increased RWT but not LV mass was associated with increased risk of in-hospital mortality after AVR [62]. Only patients with a normal LV mass index and RWT had a survival benefit when compared to those with any pattern of abnormal LV geometry [63].

Most of the available data suggest that TDI and STE derived indices of LV function might be useful for risk stratification in both symptomatic and asymptomatic patients with AS although they need validation in larger studies. A summary of the most important clinical studies addressing this issue is presented in Table 2.

**Table 2:** **Independent predictors of adverse events in patients with aortic stenosis - results of studies assessing modern echocardiographic parameters**

**Independent predictors**	**Cut-off values**	**Population**	**Adverse events**	**Follow-up**	**Reference**
- Basal longitudinal strain (STE)	−13%	- 65 asymptomatic pts with AS, AVA < 1 cm^2^, LVEF >55%	Combined end-point: re-hospitalization for any cardiac cause, aortic valve surgery, cardiovascular death within 12 months	12 months	Lafitte *et al*. [5]
- Systolic annular velocity (TDI)		- 126 asymptomatic pts with AVA ≤ 1,2 cm^2^, LVEF >55%	Combined end point: onset of symptoms; cardiac-related death; need for AVR	20.3 ± 17.8 months (median follow-up period)	Lancellotti *et al*. [64]
- Late diastolic annular velocity (TDI)					
- E/e' ratio					
- Indexed LA area					
- BNP					
- LV longitudinal deformation (STE)	- 15.9%	- 163 asymptomatic pts with AVAi < 0.06 cm^2^/m^2^; LVEF >55%	Combined end-point: cardiac death; development of significant symptoms; clinical need of AVR	20 ± 19 months	Lancellotti *et al*. [65]
- Peak aortic jet velocity	4.4 m/s				
- Valvuloarterial impedance	4.9 mmHg/ml/m^2^				
	12.2 cm^2^/m^2^				
- Indexed LA area					
- Global LV longitudinal strain (STE)	−15%	- 79 asymptomatic patients with severe AS (AVA <1 cm^2^ or transaortic jet velocity >4 m/s) and LVEF ≥ 50%	Combined end-point: cardiac death; AVR driven by symptom development	23 ± 20 months	Yingchoncharoen *et al.* [66]
- STS-PRMM					
- Aortic valve calcification score					
- AVA					
- Valvuloarterial impedance					
- E/e’ ratio (lateral annular site)	15	- 125 symptomatic and asymptomatic unoperated patients with severe AS	All cause death	1 year	Biner *et al*. [67]
- BNP	300 ng/ml				
- Global LV longitudinal strain (STE)	−15% (−12.8%^*^)	- 146 symptomatic and asymptomatic pts with mild, moderate and severe AS	All-cause mortality	median follow-up of 2.1 years	Kearney *et al*. [68]
- Age-adjusted Charlson comorbidity					
Index					
- Symptom severity class					
- Systolic peak radial strain rate (TDI)	2/s	- 32 symptomatic patients with AVR for severe AS (AVA < 1 cm^2^, LVEF 61 ± 10% )	Combined end-point: cardiovascular death, worsening of HF and limited exercise capacity	12 months	Bauer *et al*. [69]
- e'					
- E/Vp					
- Global LV longitudinal strain (STE)^**^		- 125 symptomatic pts with severe AS and LVEF >40% undergoing AVR	Combined end point: cardiovascular mortality and cardiac hospitalization due to worsening of HF	mean follow-up of 3.8 ± 1.5 years	Dahl *et al*.[70]

TDI, Tissue Doppler imaging; STE, speckle tracking echocardiography; AVA, aortic valve area; AVAi, indexed aortic valve area; LVEF, left ventricular ejection fraction; LA, left atrium; STS-PRMM, Society of Thoracic Surgeons Predicted Risk of Morbidity and Mortality; E, early diastolic transmitral velocity; e’, mitral annulus early diastolic velocity, Vp, velocity of flow propagation into the left ventricle.

^*^, this threshold provided the best combination of sensitivity (83%) and specificity (87%) for all-cause mortality.

^**^, patients were divided into 4 groups according to GLS quartiles.

Both E/e' ratio (as an expression of LV filling pressures) and GLS (as a marker of LV subendocardial function) can be easily measured in most patients with AS and have the potential of becoming useful tools for risk assessment in clinical practice. However, their incremental prognostic value over well-known haemodynamic parameters of AS severity was not clearly demonstrated. After accounting for the severity of AS, neither indexed LV mass nor any TDI derived parameter of LV function provided additional predictive information in asymptomatic patients [71]. On the other hand, low values of GLS were independently associated to increased all-cause mortality when adjusting for several established risk factors (including symptoms, LVEF and haemodynamic severity) [66,68].

Echocardiographic parameters of LV longitudinal function are strongly linked to the extent of myocardial fibrosis, which has clear prognostic implications [49]. Midwall myocardial fibrosis was associated with an 8-fold increase in all-cause mortality in patients with significant AS[60] while focal fibrosis was an independent predictor of increased perioperative risk and mortality in patients with AS undergoing surgical AVR [72].

Therefore, the echocardiographic assessment of LV longitudinal deformation allows estimation of LV subendocardial fibrotic changes and may become a tool for risk stratification in patients with significant AS. So far, the clinical utility of GLS is hindered by the lack of standardization on different echo machines and lack of specific cut-off values.

B-type natriuretic peptide (BNP) is a useful tool in risk stratification of asymptomatic patients with AS, although absolute threshold values were not adequately validated for use in clinical practice. An increase in serial BNP levels may identify a subgroup of patients with a higher degree of diastolic LV dysfunction and latent LV systolic dysfunction that may precede symptom development [73]. A serum BNP level higher than the normal upper limit for each individual patient (defined as BNP clinical activation) was a powerful predictor of long-term mortality, incrementally and independently of all baseline characteristics in a large cohort of patients with moderate and severe AS [74].

In a retrospective study in a large cohort of high risk patients who underwent transcatheter AVR low flow (LV stroke volume index < 35 ml/m^2^) was an independent predictor of cumulative all-cause and cardiovascular mortality [75]. A post-hoc analysis from the PARTNER (Placement of Aortic Transcatheter Valves) trial showed that low flow was an independent predictor of mortality in both the inoperable and high risk cohorts, whereas LV EF and transvalvular gradient were not [76]. Moreover, in patients with low-gradient severe AS and preserved EF, indexed LV stroke volume emerged as the most powerful echocardiographic parameter associated with long-term outcome, with a 20% increase in adjusted mortality risk for each 5 ml/m^2^ reduction in stroke volume index [77]. More recent data confirm that lower values of indexed LV stroke volume are independently and incrementally associated with increased mortality in these patients [78].

Dobutamine stress echocardiography provides important prognostic information in patients with low-flow low gradient AS by assessing LV contractile reserve, which aids therapeutic decision-making [40]. Incorporating measurement of peak stress longitudinal strain parameters may add incremental prognostic value [79].

### Conclusions and future perspectives

The consequences of increased afterload on the LV should always be taken into account for a comprehensive assessment of patients with AS. Beyond the conventional assessment of LV mass and ejection fraction, the assessment of LV deformation parameters (in particular STE derived GLS) and myocardial fibrosis (estimated by CMR) will probably be increasingly used in the decision making process in patients with AS in the near future. Although proper standardization and confirmatory data from large prospective studies are needed before incorporating such new parameters into practical management algorithms, their close monitoring may prevent irreversible myocardial damage and the risk related to delayed symptom reporting. Exercise echocardiography may provide incremental prognostic value by assessing both exercise-induced symptoms and changes in valve haemodynamics, LV function, and pulmonary pressures. Such an inclusive approach can aid in timing the intervention in apparently asymptomatic patients with severe AS and stratify risk in patients undergoing AVR.

In view of the fact that no single parameter of LV function predicts the optimal timing for AVR in asymptomatic patients, all available information must be considered for optimal decision-making in clinical practice.

## Abbreviations

AS: Aortic stenosis
LV: Left ventricle
LVEF: Left ventricular ejection fraction
LVH: Left ventricular hypertrophy
HF: Heart failure
RWT: Relative wall thickness
CMR: Cardiac magnetic resonance
MAPSE: Mitral Annular plane systolic excursion
TDI: Tissue Doppler imaging
STE: Speckle-tracking echocardiography
GLS: Global longitudinal strain
AVR: Aortic valve replacement
LGE: Late gadolinium enhancement
CT: Computed tomography
CAD: Coronary artery disease
BNP: B-type natriuretic peptide
